# Connecting teachers’ classroom instructions with children’s metacognition and learning in elementary school

**DOI:** 10.1007/s11409-020-09248-2

**Published:** 2021-11-20

**Authors:** Mariëtte H. van Loon, Natalie S. Bayard, Martina Steiner, Claudia M. Roebers

**Affiliations:** Department of Developmental Psychology, University of Bern, Fabrikstrasse 8, CH-3012 Bern, Switzerland

**Keywords:** Teacher instructions, Strategies, Child-centered teaching, Development, Metacognition, Elementary school

## Abstract

Many children have difficulties with accurate self-monitoring and effective regulation of study, and this may cause them to miss learning opportunities. In the classroom, teachers play a key role in supporting children with metacognition and learning. The present study aimed to acquire insights into how teachers’ cognitive and metacognitive strategy instruction, as well as teacher-directed and child-centered instructional practices are related to children’s self-monitoring accuracy, regulation of study, and learning performance. Twenty-one teachers and 308 children (2nd and 4th grade elementary school) participated. Teachers instructed a secret code task, children had to learn the match between letters of the alphabet and corresponding symbols. Teachers were observed and audio-recordings were made of their instructions. Then, children were asked to (a) make restudy selections, (b) complete a test, and (c) self-monitor test performance. Although teachers both addressed cognitive and metacognitive strategies, they more often instructed children about cognitive strategies. Further, teaching practices were more often teacher-directed than child-centered. Although there were no relations between teachers’ instructions for metacognitive strategies and children’s outcome measures, teaching cognitive strategies was positively associated with children’s performance and self-monitoring accuracy. However, teaching cognitive strategies did not predict effective restudy selections. Rather, child-centered instructions (i.e., giving children autonomy to regulate their own learning) positively predicted children’s restudy, and further, children’s self-monitoring was more accurate in classrooms where teachers more often used child-centered instructional practices. This seems to imply that not only the content of the instructions itself, but particularly the way these are given, affects children’s metacognition.

As early as in elementary school, children’s accurate metacognitive self-monitoring benefits successful self-regulation and achievement (Rinne and Mazzocco 2014; Schneider and Löffler 2016). Children with more sophisticated self-monitoring make more precise re-study decisions, allocate more study time to hard compared to easy tasks, and are more proficient at correcting errors (Destan et al. 2014; Dufresne and Kobasigawa 1989). However, many children have difficulties with accurate self-monitoring and effective regulation of study, and this may cause them to miss learning opportunities (Rinne and Mazzocco 2014; Schneider and Löffler 2016). Because elementary school children do not yet effectively use strategies to monitor and regulate their learning on their own, they need support from their teachers with this. The present study aimed to acquire insight into how teacher instructions may support children’s metacognitive accuracy (as indicated by self-monitoring and restudy selections) and learning performance.

Children in Western countries typically attend school from age six onwards, and schooling experiences are an important source for metacognitive development (Efklides 2013; Schneider and Löffler 2016). Although children’s strategy use and performance seems to be affected by teacher instructions (Grammer et al. 2013; Zepeda et al. 2019), it is unclear how variance between teachers’ instructions affect children’s metacognitive accuracy. Considering the effects of metacognition on self-regulation and learning, assessing relations between teacher instructions and children’s metacognition seems highly relevant. The present research aims to address this, by combining classroom observation measures of teachers’ instructions during a school lesson with measures of children’s metacognition and learning. In the present paper, the term instruction is being used to refer to the content of instructional talk, including teacher statements relating to specific cognitive and metacognitive strategies, as well as teacher-directed and child-centered suggestions and questions aimed to give children support with strategy use. To investigate whether and how effects of teacher instructions may depend on children’s age, teachers and children of second and fourth grade elementary school classes were compared.

## Effects of strategy instruction on learning and metacognition

Being an effective learner implies knowing about and flexibly using cognitive strategies (i.e., activities and techniques that enhance memory, comprehension and application of studied information) and metacognitive strategies (i.e., monitoring and regulating one’s learning activities; Bjork et al. 2013). Ideally, teachers instruct children about how to flexibly use cognitive and metacognitive strategies in relation to task demands and time constraints (Cornoldi et al. 2015; Dignath et al. 2008; Ornstein et al. 2010). Teachers’ instructions about cognitive and metacognitive strategies are related to children’s learning across the course of elementary school and beyond (De Boer et al. 2018; Donker et al. 2014; Grammer et al. 2013; Ornstein et al. 2010).

Although most research focuses on teaching of single strategies, research by Ornstein et al. (2010) showed how teachers’ classroom instructions about use of multiple cognitive and metacognitive strategies affected children’s learning in the short and longer term. They audio-recorded and coded teacher instructions to investigate variability between teachers’ instructions of strategies such as making associations, rehearsal and testing. Teachers varied considerably in the extent to which they instructed about such mnemonic techniques. Importantly, students of the so-called high mnemonic teachers, who more often taught about cognitive and metacognitive strategies, showed better memory strategies and performed better on memory and problem-solving tasks. Further, several years later, effects of teaching about cognitive and metacognitive strategies and use of memory-relevant language still seemed to affect children’s memory strategies and study skills, indicating that the high mnemonic teaching equipped children with strategic skills that were transferable to other tasks. However, all teachers were instructing with use of their own learning materials, which made it difficult to compare teachers.

With an experimental study, Grammer et al. (2013) aimed to directly assess the relation between teacher instructions for strategies and children’s cognitive strategy use and learning. The task content (a science task in which children learned about movement) was kept constant between teachers, and the subsequent assessment of children’s factual and procedural knowledge was directly related to the instructed task. All teachers received scripted lessons to teach about the task content. For one group (the memory rich instructional condition), these lessons instructed about how to use memory strategies, and teachers asked children metacognitive questions. In the other group (the low memory condition), teachers did not explicitly instruct about the use of cognitive and metacognitive strategies. Results of this study suggested that the content of teacher instructions affects children’s strategy knowledge, strategy use, and performance. Children who were exposed to memory rich teaching showed better strategic knowledge and application of strategies, and had higher outcomes on memory and problem-solving tasks at a post-test. One month after the experiment, benefits of memory-rich instruction were still visible on delayed strategy assessments and memory tests. However, since teachers in the memory-rich condition all taught the same scripted lessons, it remains unanswered how individual differences and variability between teachers are related to children’s learning in a more naturalistic instructional setting.

A study with adolescents by Zepeda et al. (2019) brings more insight into how individual differences between teachers in metacognitive talk may affect sixth to eight grade students’ growth in math performance. Teachers were videotaped when teaching about ratio and proportion, and teacher talk was compared between classes with low and high performance growth on math tests. Teachers’ instructions for metacognition were related to growth in student performance; in classes with high growth in math scores, teachers instructed more about monitoring and evaluation than in classes with low growth in performance.

Moreover, in addition to affecting cognitive strategy use and task outcomes, teacher instructions may affect children’s use of metacognitive strategies. Leidinger and Perels (2012) provided one group of teachers with a training on how to teach elementary school children about self-regulation strategies when learning math (including goal-setting and planning strategies). A control group of teachers did not receive such a training. All children completed diaries during the duration of the training (6 weeks) and they completed a questionnaire about their self-regulated learning activities after the training. The students of the trained teachers reported that they more often used goal-setting and planning strategies in comparison to the control group. However, although this research indicates relations between teacher instructions and children’s metacognitive strategy use, children’s actual metacognitive accuracy was not assessed. Leidinger and Perels (2012) only investigated children’s self-reported use of learning strategies, even though such self-reports may not correlate with actual behavioral measures (Cromley and Azevedo 2006).

In sum, several studies document relations between teachers’ cognitive and metacognitive strategy instructions and children’s strategy use and achievement, although for elementary school children, it remains unclear how variability among teachers’ strategy instructions is related to learning. Moreover, to our knowledge, no research investigated how teacher instructions are related to children’s metacognitive accuracy. We therefore aim to acquire insight into the connections between teachers’ instructions about cognitive and metacognitive strategies and children’s learning and metacognition. Importantly however, when learning about how to engage in metacognition, children may not only need to learn about strategies, but also need to have opportunities to practice strategy use during self-regulated learning activities (Coffman et al. 2008; Dignath and Buttner 2008; Zepeda et al. 2019). Therefore, in addition to investigating the content of teachers’ strategy instructions, we also address to what extent teachers provide children with opportunities to apply strategies.

## Teacher-directed and child-centered teaching practices

Generally, when instructing children about how to use strategies, instructions can be categorized as being teacher-directed or child-centered (Pakarinen and Kikas 2019; Stipek and Byler 2004). Teacher-directed teaching entails directly instructing children how to work on a task, and teaching learning strategies through explanation and modelling. For instance, during teacher-directed instruction about association strategies, a teacher might explicitly tell and model how associations between task items can be built. When using child-centered teaching practices, teachers structure learning situations to encourage children to practice strategies in a self-regulated manner, so they can construct their own insights about how and when to use these (Cadima et al. 2015; Collins 1996; Lerkkanen et al. 2016). Rather than telling children explicitly how to work and when to use specific strategies, child-centered instruction is aimed at promoting children’s autonomy (Lerkkanen et al. 2016). The teacher co-regulates student learning by guiding the learning process (typically through questioning rather than through giving directives) and provides assistance on an as-needed basis (often based on help-seeking by students).

Research concerning effects of teacher’s instructional practices on children’s cognitive and metacognitive development is still in its early stages, and it is yet an open question how child-centered and teacher-directed instructional practices can be most beneficial. For metacognition, teacher-directed instruction seems to focus children’s attention on task-relevant information and can help children with organization (Bonawitz et al. 2011; Stright and Supplee 2002). At the same time, in comparison to child-centered instruction, the teacher-directed approach may also lead to less behavioral engagement (Cadima et al. 2015), self-regulated task exploration (Bonawitz et al. 2011), and engagement in self-monitoring activities (Stright and Supplee 2002). Rather than telling students exactly what to do with directive statements, inviting children to think about questions may support them to engage in metacognition (Zepeda et al. 2019).

For learning achievement, effects of teaching practices are debated. Some studies show a benefit of child-centered instructional practices on performance across different learning domains (Lerkkanen et al. 2016). However, particularly for weaker students, there are indications that teacher-directed instruction has advantages for performance (Kistner et al. 2010; Zepeda et al. 2019). To complicate matters, instructional practices seem to depend on children’s development. However, the few existing studies on effects of teacher instructions seldom considered the combination of contextual (i.e., classroom) and developmental determinants of children’s metacognition and learning, even though it may be necessary to consider these factors in combination.

## Relations between development of metacognition and teacher instructions

In the present study, we specifically focus on children’s metacognitive accuracy, i.e., their ability to accurately self-monitor their performance and to effectively select not-yet-learned items for further study. These metacognitive accuracy measures indicate components of procedural, rather than declarative metacognition (i.e., self-reported task and strategy knowledge; Schneider and Löffler 2016).

Approximately until the age of eight, children’s ability to accurately self-monitor their learning, and to differentiate between correct and incorrect performance, becomes increasingly reliable and slowly generalizes from perceptual tasks to more complex learning and memory materials (for reviews see Roebers 2014, 2017; Schneider and Löffler 2016). Around age eight, children are generally able to discriminate between correct and incorrect responses (although they remain overconfident for errors) when working with relatively simple materials such as learning of paired-associates and vocabulary (Roderer and Roebers 2010). The ability to accurately monitor learning of more complex materials, such as judging comprehension of cause-and-effect relations in texts, does not seem to develop until children are approximately 12 years old, and these skills keep developing at least until late adolescence (De Bruin et al. 2011; Schneider and Löffler 2016). Furthermore, children’s ability to regulate learning continues to develop across the elementary school years and beyond (Metcalfe and Finn 2013; Schneider and Löffler 2016). For instance, 12-year-old elementary school children seem better able than 10-year-old children to use their monitoring judgments to strategically select difficult or not-yet-learned items for restudy (Van Loon et al. 2013).

Children’s cognitive and metacognitive development is not only an effect of biological maturation processes, but also of contextual influences (Crone and Steinbeis 2017; Efklides 2013; Roebers et al. 2019). Continuous changes in the child’s environment are thought to be one of the main factors driving children’s development. In elementary school, the classroom is one of the most important learning environments for children. There, children’s learning is co-regulated through interactions with teachers and peers (Salonen et al. 2005). Teachers ideally support children by giving instructions about how to use cognitive and metacognitive strategies, and by designing learning tasks and learning environments that enable children to practice self-monitoring and self-regulation of learning (Dignath and Büttner 2018).

Given the development-related improvements in cognitive and metacognitive strategies across the elementary school years, teachers continuously need to adapt their instructional content and practice to children’s skills (Collins et al. 1988). Strategy teaching is a scaffolding process; child-centered and teacher-directed instruction are employed at different stages of the learning process (Pressley et al. 1992). Younger and weaker students mainly seem to profit from teacher-directed instruction; because young elementary school children may not be able to self-regulate their learning and have to learn how to cope with autonomy, teacher instructions may predominantly be direct and detailed. When teaching about cognitive and metacognitive strategies, teachers would then model how to use these, and explicitly explain when and how to use strategies (Collins et al. 1988; Veenman 2007). Older and stronger learners seem to profit more from child-centered methods; presumably because they are better able to self-regulate their learning. Indeed, Zepeda et al. (2019) showed that for weaker students, teachers more often explicitly modelled strategy use, whereas in the higher-achieving classes, teachers rather used questioning and prompting supports.

In sum, with increasing age, experience and competence, children are expected to internalize the strategies being taught and to take more control over their own learning (Collins et al. 1988; Pressley et al. 1992). Over the course of elementary school, teachers may therefore gradually switch from using teacher-directed practices to use of more child-centered instructional approaches (Perry et al. 2015). That is, the content of strategy instruction, as well as the balance between teacher-directed and child-centered instructions seems to depend on children’s development. However, to our knowledge, it remains unknown to what extent the content of strategy instruction as well as instructional practices have differential effects on performance and procedural metacognition for younger and older children. These issues are investigated in the present study, by comparing second and fourth grade students.

## The present study

The main aim of the present research is to investigate how teachers’ instructions affect children’s procedural metacognition and learning. RQ1 addresses (a) how often teachers instruct about cognitive and metacognitive strategies; (b) how often they use teacher-directed and child-centered practices, and (c) whether cognitive and metacognitive strategy instructions and teacher-directed and child-centered teaching practices differ between second and fourth grade elementary school teachers. RQ2 addresses how teachers’ strategy instructions and instructional practices are related to children’s (a) memory performance and (b) metacognitive accuracy. RQ 3 investigates whether relations between teacher instructions and children’s memory and metacognition may depend on children’s age (i.e., grade level.)

To be able to compare the participating teachers and children with each other, we controlled for the domain to be taught, such that all teachers instructed about the same task content, for the same duration of time. Specifically, all participating teachers instructed a secret code task, for which children had to study associated pairs of letters and symbols. To reliably picture teachers’ behavior with regard to instructional content and practices, instructions were audio recorded during the classroom lessons. Subsequently, we acquired data with use of validated cognitive and metacognitive performance measures for children. For the secret code task children had to strategically associate and memorize pairs. For such associative learning tasks, it is well established that most elementary school children, although overconfident for errors, are able to discriminate between correct and incorrect performance. For this type of task, developmental differences are mainly apparent for measures of regulation (Schneider and Löffler 2016).

To assess the direct, immediate relation between teacher instructions and children’s outcomes, measurement of children’s task performance and metacognition took place right after the teacher instruction phase. That is, after classroom instruction by teachers, children selected task items for restudy (to measure effectiveness of regulation), completed a test (measuring memory performance), and judged their confidence in each of their test responses (to assess self-monitoring accuracy).

In line with previous classroom observation studies (Dignath and Büttner 2018; Ornstein et al. 2010; Spruce and Bol 2015), we expected that teachers would more often focus on cognitive strategies than on metacognitive strategies and processes. Although we expected more teacher-directed than child-centered teaching practices for both grades (cf. Alford et al. 2016; Stright and Supplee 2002), in line with Collins et al. (1988), we expected more child-centered teaching practices for fourth than for second graders.

Furthermore, because research seems to indicate that classroom instructions for cognitive and metacognitive strategies positively affect children’s learning on a variety of tasks (Ornstein et al. 2010; Zepeda et al. 2019), we expected that cognitive and metacognitive strategy instructions would be positively related to children’s memory performance. The most important novelty is that this research addresses relations between teacher instructions and children’s metacognitive accuracy. Furthermore, research findings on how teacher-directed and child-centered instructions are related to performance and metacognition are mixed. Moreover, it is yet unclear how relations between teacher instructions and children’s metacognition and learning may depend on children’s age. To clarify these issues, these are addressed exploratively.

## Methods

### Participants

Participants were 21 public elementary school teachers (19 females) and 308 children; 139 children were second graders, *M* age = 7.6 years (*SD* = .50), 48.9% girls, and 169 were fourth graders, *M* age = 9.7 years (*SD* = .57), 47.3% girls. Of the children, 75.5% reported to be native [XXX] speakers. All participating children were familiar with following school instructions in the [XXX] language. The children in the sample were part of a larger longitudinal study with three data waves (and seven measurements) over the course of 1 year, assessing metacognitive development for a core group of 324 children.

All participating teachers had a bachelor’s degree in education, and teaching experience ranged from 10 months to 40 years. The size of their class ranged from 15 to 24 children.

Testing took place in publicly funded schools in [information withheld]. For all children, informed consent was received prior to testing. The study was realized in accordance with the APA ethical principles and the declaration of Helsinki, and the Faculty Ethic Review Board of [information withheld] approved the research project.

### Materials and procedure

**Fig. 1:**
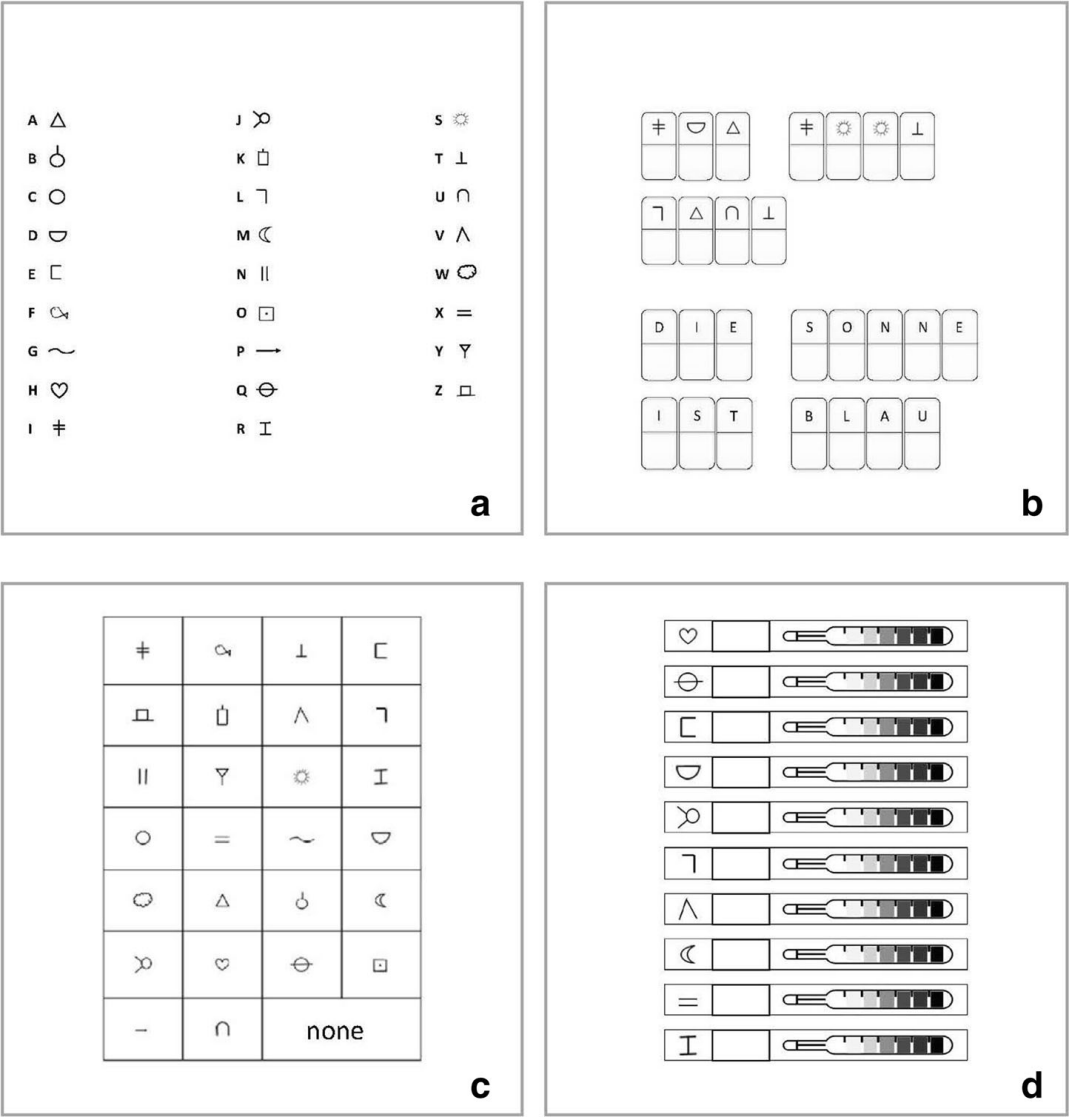
Materials. **a**. The secret code task. **b**. An example of a reading and a writing task, as presented in the practice booklet. **c**. A grid presenting the items to indicate selections for restudy. **d**. A part of the secret code test and the thermometer on which children indicated with confidence judgments how certain they were each response was correct

Participating teachers were asked to work with their class on a cryptography (i.e. a secret code) learning task for 30 min. As shown in Fig. 1a, for the secret code task, 26 letters of the alphabet were matched to 26 corresponding symbols.

All materials were developed for the purpose of this study; to ensure that the materials would be suitable for both age groups, these were piloted in five classrooms with children (*n* = 93) in the same age range as the participants in the present study (2 s grade classes, *n* = 41; three fourth grade classes, *n* = 52). A subset of the items was associated based on visual similarity of the shape of the letter and symbol; a subset was associated based on a match between the letter and the first letter of the symbol (e.g. the letter “F” was related to the symbol fish), and for a subset of items, letters and symbols were not obviously related. Based on the pilot study, materials were made somewhat more difficult for fourth graders than second graders, by presenting them with fewer items that were clearly associated (14 for fourth grade versus 20 for second grade), and thus more items that were not obviously related (12 for fourth grade versus six for second grade), to prevent ceiling effects in performance.

Further, materials consisted of a practice booklet in which children could practice reading and writing the secret code. This booklet consisted of four reading tasks and five writing tasks. Figure 1b shows an example of a reading and a writing task. The reading tasks asked children to read the secret code; these consisted of a short word (task 1), a longer word (task 2), a short sentence (task 3) and a longer sentence (task 4) written in the secret code, with space to write down the meaning of these words and sentences. For the writing tasks, children had to write the secret code; these tasks consisted of a short word (task 1), a longer word (task 2), a short sentence (task 3), a longer sentence (task 4), and an assignment to write a secret code message to a classmate (task 5). The tasks in the practice booklet were made more difficult for fourth graders, by asking them to read and write longer words and sentences.

One week in advance of the lesson, the teachers received a sheet with the secret code items, so they could prepare their lesson. With an information letter, they were informed that the aim of the lesson was that children would memorize the secret code items, and that they could teach for 30 min in their own way. Further, the letter informed them that they would also receive a practice booklet, and that they could use additional materials of their own choice for teaching if they liked to do so. As well, they were told that after 30 min of teaching, the observers would organize the last part of the lesson, in which children had to complete the test. Further, before starting the lesson, the teachers received a poster on which the letter-symbol pairs were printed in A0 format.

When teaching about the secret code in the classroom, the teachers were observed by two to three trained observers, who completed detailed field notes throughout the observation period, for example, whether children were accomplishing activities in groups or individually, the process of the lesson, and how seating was arranged in the classroom. Furthermore, all teachers wore a recording device, to make audio-recordings of their instructions.

At the start of the lesson, an alarm was set to go off after 30 min. The teacher was told that time could be used for instruction and student practice until the alarm would go off, and that then, one of the observers would further instruct the children about the test phase.

After 30 min, all materials that were used for the teaching of the secret code were collected by the observers. Then the test booklets were handed out to the children, with a colored marker. These booklets consisted of a page on which children could make restudy selections (as shown in Fig. 1c); all symbols were presented in a grid, with instructions to mark the symbols children would like to restudy. Also, a ‘none’ option was presented in case children would decide not to restudy any symbol. Note that children could not actually restudy the selected items (because this would then affect test performance).

After making restudy selections, children found a page on which they read that they should stop and raise their hand. After a child raised the hand, the experimenter went to the table of that particular child, collected the colored marker, and gave the child a pen (this way, it was ensured that children could not change their restudy selections after taking the test). Then, the experimenter flipped the page so the test could be started. Figure 1d shows a part of the test and the self-monitoring task. For the test, children were presented with the secret code symbols for which they had to write down the matching letter. Next to each text answer, a 7-point cold-hot thermometer (as used by Koriat and Shitzer-Reichert 2002) was printed, on which they could indicate with confidence judgments (CJs) how certain they were that the given response was correct. For the test booklet, two different versions were handed out to prevent children from copying each other’s test answers; the only difference between these was the order of item presentation.

### Coding of teacher instructions

The audio recordings that were made during the 30 min of classroom instruction were transcribed; the transcriptions were then coded with MAXQDA software. Although all interactions between teachers and the children were transcribed, the coding only focused on teachers’ instructional language. In line with Dignath-van Ewijk et al. (2013), any teacher utterance for which it could be assumed that students would learn strategic behavior was coded as a strategy instruction. Specifically, teacher statements relating to specific cognitive and metacognitive strategies, as well as teacher suggestions and questions aimed to give children support with strategy use were coded. Appendix Table 7 presents an example of a part of a transcript and the coding.

As described in more detail below, coding of the transcripts was based on existing observation instruments to code teachers’ strategy instructions. However, not all codes from existing instruments were applicable to the present research. Moreover, when observing classroom teaching during the pilot study, further coding categories emerged. Our final coding scheme underwent review by three co-raters, who extensively discussed, based on pilot study data, how to define the teacher behaviors and how these could be meaningfully coded. After coding transcripts from our actual study with use of this coding scheme, four of the transcripts were coded by two independent raters. MAXQDA software was used to compute interrater reliability, this ranged from 79.5% to 88.5% (Kappa .78 to .88), indicating good interrater agreement among the coders (Landis and Koch 1977).

Table 1 shows the used codes. Teacher instructions were coded in multiple rounds; first, we coded the transcripts for instructions for strategies (cognitive and metacognitive), and then, teachers’ instructional language was coded as teacher-directed or child-centered instructional practice. Note that parts of teacher instructions in the transcripts could receive multiple codes, and that therefore, codes could overlap. Examples of this are given in a sub-part of a coded transcript in Appendix Table 7, and further, Appendix Table 8 shows for each code how, in case of overlap, this code co-occurred with other codes.

**Table 1:** Coding of strategy instructions and instructional practices

Instructions	
Instructions for Cognitive Strategies	
Making associations	Teacher gives an example of an association between a symbol and a letter or motivates children to consider associations.
Self-testing	Teacher asks children questions to assess their knowledge or tells children to test themselves.
Other cognitive strategies	Teacher uses other strategies to teach about the task, for instance, drawing strategies and using objects.
Instructions for Metacognitive Strategies	
Setting and monitoring task goals	Teacher defines goals or reminds students to consider end- or sub goals of the lesson.
Planning time and use of task materials	Teacher addresses the time planning or planning how to work with available materials, or asks students to think about planning time and material use.
Self-monitoring task progress	Teacher tells children to evaluate their task progress, or asks whether students understood the task and whether they have questions.
Monitoring item difficulty	Teacher distinguishes between easy and difficult items or asks students to consider item difficulty.
Attending to errors/mistakes	Teacher discusses actual and potential errors or misconceptions.
Evaluation of strategies	Teacher explains (dis)advantages of used strategies or asks students to consider (dis)advantages of the strategies they use.
Instructional Practices	
Teacher-directed practice	Teacher tells and/or modeled how to use a cognitive or metacognitive strategy. Teacher tells the specific goals and standards which should be reached. Teacher tells children which part of the task they should work on.
Child-centered practice	Teacher asks children to share ideas and interpretations about how strategies can be used. Teacher asks children to practice use of strategies themselves. Children are told that they can decide themselves what to work on and how to work on this.

#### Strategy teaching

For strategy teaching, in line with Dignath-van Ewijk et al. (2013), Donker et al. (2014), Kistner et al. (2010), we coded cognitive and metacognitive strategies that were taught by the teachers. Furthermore, based on research showing effects of strategy use on children’s learning, we defined subcategories within these two areas more specifically. *Cognitive strategies* directly refer to information processing of the learning task children are working on. Due to the nature of the task (a paired-associate memory task), only a subset of cognitive strategies could be observed in the present study (for instance, teachers never taught summarization strategies). The most often observed cognitive strategies were *making associations* and *self-testing*. Elementary school children seem to rarely use such association strategies and group items based on relatedness by themselves, but they seem well able to apply this strategy and improve memory when they are taught about this (Bjorklund et al. 1990). Also teaching about self-testing seems very meaningful, since this cognitive strategy benefits learning and memory across a range of learning tasks (Pyc and Rawson 2010). Although some further cognitive strategies were observed and coded, such as visualization strategies, instruction of such strategies only occurred very rarely. Therefore, these were coded as *other cognitive strategies*.

*Metacognitive strategies* were defined as strategies meant to support learners to monitor and regulate their cognitive task processing. The coding tool developed by Spruce and Bol (2015), and the ATES observation tool (Assessing how Teachers Enhance Self-regulated learning; Dignath-van Ewijk et al. 2013) were used as guides to code for metacognitive strategy teaching. These coding instruments are based on the model of self-regulated learning by Zimmerman (2011), and align with the three phases of self-regulated learning: Forethought, performance, and self-reflection.

The codes *setting and monitoring task goals* and *planning time and use of task materials* can be classified under the forethought phase. These codes were selected because studies with children indicate that teachers’ instructions to consider learning goals and to plan learning actions have strong effects on learning (Donker et al. 2014).

The codes *self-monitoring task progress* and *monitoring item difficulty* can be classified under the performance phase. It is clear that teachers should support students to self-monitor whilst working on learning tasks; and this code is included in several classroom observation instruments (Dignath-van Ewijk et al. 2013; Spruce and Bol 2015). Moreover, during the observations in the context of our pilot study, we also discovered that teachers regularly instructed about monitoring item difficulty, and this was considered a meaningful strategy related to teaching for metacognition during the performance phase. Elementary school children seem to have some awareness that more difficult items are harder to memorize (Koriat and Ackerman 2010a, b), but often do not use these insights by themselves.

The codes *attending to errors/mistakes* and *evaluation of strategies* can be categorized under the self-reflection phase. When teachers support children to identify when they are struggling and making errors, this can give children the opportunity to improve learning (Bjork et al. 2013). Further, teaching children about how to evaluate the value and the effectiveness of the strategies they used has been identified as very valuable for learning (Dignath and Büttner 2018; Ornstein et al. 2010).

#### Coding of instructional practices

With coding of instructional practices, we examined whether instructions were provided in a *teacher-directed* or in a *child-centered* manner. The ECCOM observation tool (Early Childhood Classroom Observation Measure; Stipek and Byler 2004) was used as a basis. The code *teacher-directed* was used when the teacher dominated the instructional conversation, explicitly told or modeled how strategies should be used, instructed which specific goals and standards had to be reached, or explicitly told children which part of the task they had to work on. Teachers’ instructional language was coded as *child-centered* when the teacher solicited children’s ideas and interpretations about how strategies could be used, when children were asked to practice use of strategies themselves, and when children were told that they could decide themselves what to work on and how to work.

### Analyses

#### Teacher instructions

For every teacher, cognitive strategies (making associations, self-testing, and other cognitive strategies) were collapsed, to create a variable indicating the overall frequency count of teaching for cognitive strategies. As well, metacognitive strategies (setting and monitoring task goals, planning time and use of resources, self-monitoring task progress, monitoring item difficulty, attending to errors/mistakes, and evaluation of strategies) were collapsed, to acquire a variable indicating the overall frequency of teaching for metacognition. Appendix Table 6 shows the sub-codes underlying the overall frequency coding for strategy instructions. Due to the small sample size of teachers, we compared instructions for the two age groups with non-parametric tests.

#### Children’s performance and metacognition

To investigate children’s performance on the secret code test, for each item, the response was scored as 1 (correct) or 0 (incorrect response or omission). Then, for each child, mean performance was calculated as the percentage of correct task responses.

To investigate the effectiveness of children’s restudy selections, we investigated the percentage of items that were selected for correct and incorrect test responses separately. For effective learning, children should restudy the less well-learned items for which performance is incorrect, rather than task items for which responses are correct (Nelson et al. 1994). To investigate the effectiveness of restudy, we calculated a difference measure between restudy selections for incorrect and correct responses. A larger difference measure indicates that children more effectively differentiated between incorrect and correct task items in their restudy selections.

To assess self-monitoring accuracy, for each child, mean CJs (range 1–7) were calculated for correct and incorrect task responses separately. In line with Destan and Roebers (2015), per child, a difference measure was calculated between CJs for correct and incorrect task responses. A larger difference score indicates that children more strongly discriminated between correct and incorrect task responses when making CJs (indicating more accurate self-monitoring).

#### Relations between teacher instructions and children’s memory, restudy, and self-monitoring accuracy

Firstly, *F*-test were conducted to address whether variance on the individual level could be explained by variance on the classroom level (as recommended by Snijders and Bosker 2012). To account for the nested structure of the data, linear mixed model analyses were used to analyze the effects of instructional content (i.e., teaching for cognitive and metacognitive strategies) and instructional methods (teacher-directed and child-centered teaching) on performance, restudy accuracy, and the accuracy of CJs for both grades. With these analyses, we can address our hypotheses concerning the effects of teacher predictors (instructional content and practices) and the predictor grade on dependent variables measured per child (performance and metacognition). Individual children were entered at level 1, teachers/classes at level 2, and grade at level 3.

The variables indicating the frequency counts of teaching for cognitive strategies, teaching for metacognitive strategies, and teacher-directed and child-centered practices, were used as predictors for children’s outcome measures (i.e., memory performance, restudy effectiveness, and self-monitoring accuracy). In all models, grade was included as a fixed factor; the reference group was the fourth grade to which the second grade was compared. Further, in all regression models, interactions between grade and the teacher-level predictors were included; these interaction effects are only interpreted when significant.

Accordingly, for the effects of cognitive and metacognitive strategy instructions on children’s outcome measures, the regression model is:

1 $$ {\varUpsilon}_{ijk}={b}_0+{b}_1 Grade+{b}_2{CognitiveStrategyInstruction}_{jk}+{b}_3{MetacognitiveStrategyInstruction}_{jk}+{b}_4{\left( Grade\ CognitiveStrategyInstruction\right)}_{jk}+{b}_5{\left( Grade\ MetacognitiveStrategyInstruction\right)}_{jk}+{\varepsilon}_{ijk} $$

For effects of teacher-directed and child-centered instructional practices on children’s outcome measures, the regression equation is:

2 $$ {\varUpsilon}_{ijk}={b}_0+{b}_1 Grade+{b}_2{TeacherDirectedPractices}_{jk}+{b}_3{ChildCenteredPractices}_{jk}+{b}_4{\left( Grade\ TeacherDirectedPractices\right)}_{jk}+{b}_5{\left( Grade\ ChildCenteredPractices\right)}_{jk}+{\varepsilon}_{ijk} $$

In these equations, *ϒ* indicates the outcome measure for children’s (task performance, restudy effectiveness, and self-monitoring accuracy, respectively), *i* indicates the child level, *j* indicates the teacher level, and *k* indicates the grade level.

All predictors and outcome measures were *z*-standardized. For significant effects, the estimated value of the standardized regression coefficient for the fixed effects is reported as a measure of effect size.

## Results

In this section, we first describe how teachers in both grades taught the secret code tasks, and specifically, how often they instructed for cognitive and metacognitive processes, and how often instructions were teacher-directed vs. child-centered. We then describe findings on children’s performance and metacognition. Finally, to acquire insight into the relation between classroom instructions and metacognition and learning, we connect teachers’ classroom instructions to children’s individual outcome measures.

### Teacher’s strategy instructions and instructional practices

Table 2 shows the number of times cognitive and metacognitive strategy instructions and child-centered and teacher-directed instructional practices were coded in the transcripts of teachers’ instructional language for both grades. This table shows that teachers varied extensively (as indicated by the range) in the number of times they talked about cognitive and metacognitive strategies, and the number of times they practiced teacher-directed and child-centered teaching. A Wilcoxon signed rank test showed a non-significant trend that teachers more often discussed cognitive strategies than metacognitive strategies, *z =* −1.72, *p* = .005. Although teachers used a combination of teacher-directed and child-centered instructions, a Wilcoxon signed rank test showed that teaching was most often teacher-directed, *z* = −2.87, *p* = .004. Kolmogorov-Smirnov tests did not show differences between grades in the number of instructions for cognitive (*D* = .567, *p* = .905) and metacognitive strategies (*D* = .504, *p* = .961), and in how often teachers used teacher-directed (*D* = .630, *p* = .822) and child-centered instructions (*D* = .756, *p* = .617).

**Table 2:** Teachers’ strategy instructions and instructional practices

	Grade 2 Teachers			Grade 4 Teachers			Overall
	Mean	SD	Range	Mean	SD	Range	Mean
Cognitive Strategy Instructions	47.11	23.56	24–86	49.75	29.43	15–97	48.62
Metacognitive Strategy Instructions	36.33	17.01	16–64	34.83	15.16	17–62	35.48
Teacher-Directed Practices	55.78	16.83	35–91	51.83	23.17	24–89	53.52
Child-Centered Practices	37.67	11.95	24–57	35.08	20.65	7–66	36.19

*Note.* The frequency count, SD, and the range cognitive and metacognitive strategy instructions and teacher-directed and child-centered practices were coded per teacher for both grades

### Children’s performance and metacognition

Table 3 shows performance for the secret code task. Performance was higher for the fourth than the second graders, *t*(304) = 3.60, *p* < .001.

**Table 3:** Children’s performance, restudy selections, and confidence judgments

	Performance	Items selected for restudy (in %)	Mean Confidence Judgments (range 1–7)
Grade 2	85.64% (15)	14.03% (24)	6.16 (1.0)
Grade 4	90.44% (13)	12.81% (21)	6.48 (.8)

*Note.* Mean performance, the overall percentage restudy selections, and the overall magnitude of confidence judgments for both grades. Standard deviations of the mean in parentheses

**Fig. 2:**
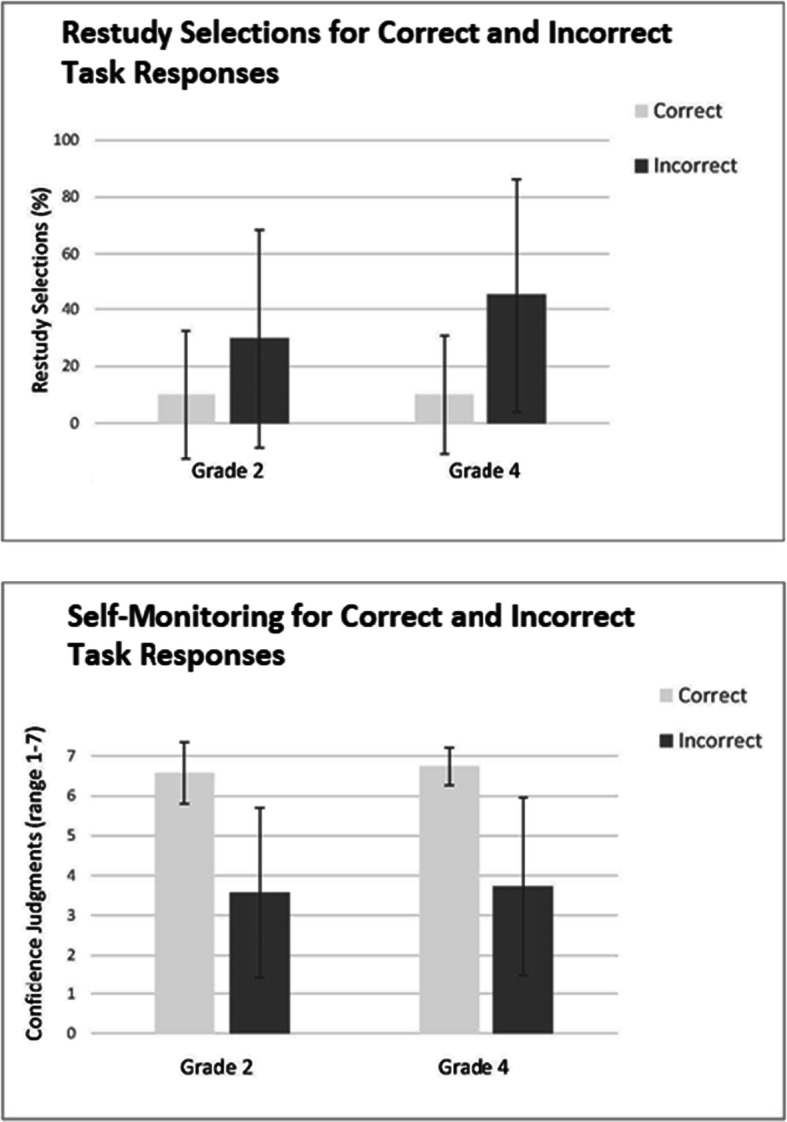
Children’s metacognitive accuracy. The upper panel shows restudy selections for correct and incorrect task responses. Children discriminated between these response types when making restudy selections, indicated by the finding that they more often selected incorrect than correct responses for restudy. The lower panel shows self-monitoring accuracy, as measured with confidence judgments for correct and incorrect task responses. Children discriminated between these response types, as indicated by the finding that they were more confident for their correct than incorrect responses. Error bars indicate the standard deviation of the mean

Further, Table 3 shows the percentage of items selected for restudy for both age groups; the overall percentage of restudy selections did not differ between the grades, *t*(306) = .46, *p* = .64. However, for effectiveness of restudy (as depicted in Fig. 2), analyses show that fourth graders’ restudy selections were more effective than second graders’ selections, *t*(206) = 2.74, *p* = .007. That is, fourth graders most accurately differentiated between learned and not-yet learned items. Follow-up analyses showed that this more effective restudy for fourth graders was due to the fact that they selected more items for which responses were incorrect for further study than second graders (45.10%, *SD* = 21.54 and 29.79%; *SD* = 24.34 for fourth and second grade, respectively).

For self-monitoring accuracy, overall mean CJs are shown in Table 3. Mean CJs were higher for fourth than for second graders, *t*(302) = 30.3, *p* = .003, indicating that overall, fourth graders were more confident that responses were correct. Further, to assess children’s ability to accurately discriminate between correct and incorrect responses when making CJs, CJs were compared for both response types. As shown in Fig. 2, for both grades, CJs were higher for correct than for incorrect responses, indicating that children were able to discriminate when self-monitoring their performance. The ability to discriminate between correct and incorrect responses did not differ between grades, *t*(193) = .09, *p* = .93.

### Relations between teachers’ instructions and children’s performance and metacognition

#### Effects of teaching cognitive and metacognitive strategies

With use of linear mixed model analyses, we investigated whether cognitive and metacognitive strategy instruction, as measured on the teacher level, predicted performance and metacognition on the child level. Preliminary analyses showed that there were significant classroom effects on children’s performance (*F* 1, 20 = 11.13, *p* < .001; ICC = .43), effective restudy (*F* 1, 20 = 2.51, *p* = .001; ICC = .13), and monitoring accuracy (*F* 1, 20 = 3.09, *p* < .001; ICC = .19). Table 4 presents the standardized regression coefficients and significance levels for the effects of strategy teaching on task performance, restudy effectiveness, and self-monitoring accuracy. Firstly, as shown in Table 4a, the multilevel analysis showed that the frequency of teaching for cognitive strategies was a positive predictor of children’s performance. Moreover, there was a significant interaction between grade and teaching cognitive strategies, such that teaching of cognitive strategies was more strongly related to performance in fourth than in second grade. There was no main effect of teaching for metacognitive strategies on performance.

**Table 4:** Multilevel regression of strategy instructions on children’s performance and metacognition

a			
Predictors for Children’s Task Performance	*β* (standardized)	SE	*p* value
Intercept	.41	.05	< .001
Grade (reference group 4th grade)	−.32	.08	< .001
Cognitive Strategy Instructions	.20	.05	< .001
Metacognitive Strategy Instructions	.03	.05	.542
Grade X Cognitive Strategy Instructions	−.15	.07	.028
Grade X Metacognitive Strategy Instructions	.10	.08	.208
b			
Predictors for Children’s Restudy Effectiveness	*β* (standardized)	SE	*p* value
Intercept	.04	.09	.635
Grade (reference group 4th grade)	−.42	.12	.001
Cognitive Strategy Instructions	−.24	.08	.010
Metacognitive Strategy Instructions	−.16	.09	.091
Grade X Cognitive Strategy Instructions	.14	.10	.137
Grade X Metacognitive Strategy Instructions	.08	.12	.471
c			
Predictors for Children’s Self-Monitoring Accuracy	*β* (standardized)	SE	*p* value
Intercept	.22	.10	.034
Grade (reference group 4th grade)	−.10	.14	.466
Cognitive Strategy Instructions	−.25	.08	.028
Metacognitive Strategy Instructions	−.12	.10	.266
Grade X Cognitive Strategy Instructions	−.26	.13	.054
Grade X Metacognitive Strategy Instructions	−.07	.16	.659

*Note.* The standardized regression coefficients, standard errors of the standardized regression coefficients, and *p* values indicating the significance levels of the regression coefficients for the effects of teachers’ strategy teaching on children’s (a) task performance, (b) restudy effectiveness, and (c) self-monitoring accuracy. *p < .05; **p < .01

As indicated by Table 4b, analyses for restudy effectiveness show a significant effect of teaching cognitive strategies. However, the direction of this effect seems to indicate that when teachers more often talked about cognitive strategy use in the classroom, this was negatively related to the effectiveness of restudy, such that children less accurately differentiated between incorrect and correct responses when making restudy selections. There was no effect of teaching metacognitive strategies on restudy effectiveness. Further, the main effect of grade indicates better restudy selections for the fourth grade, confirming findings on age differences in restudy as found with the above-mentioned analyses addressing the children-level only.

For self-monitoring accuracy, as shown in Table 4c, there was a significant effect of teaching cognitive strategies, such that more teaching for cognitive strategies was related to better self-monitoring, as indicated by the extent to which children’s CJs discriminated between correct and incorrect performance. There was no effect of teaching metacognitive strategies on self-monitoring accuracy, and there was no main effect of grade.

#### Effects of teaching practice on children’s performance and metacognition

A final aim of the present research was to investigate effects of teacher-level instructional practices (teacher-directed and child-centered) on children’s performance, restudy effectiveness, and self-monitoring accuracy. Regression coefficients and significance levels are presented in Table 5. Table 5a shows that both the number of teacher-directed instructions, as well as the number of child-centered instructions were positively related to performance. The effect of grade indicates that performance was lower for second than for fourth graders.

**Table 5:** Multilevel regression of instructional practices on children’s performance and metacognition

a			
Predictors for Children’s Task Performance	*β* (standardized)	SE	*p* value
Intercept	.42	.05	< .001
Grade (reference group 4th grade)	−.29	.08	.001
Teacher-Directed Practices	.09	.04	.037
Child-Centered Practices	.09	.04	.024
Grade X Teacher-Directed Practices	.10	.08	.201
Grade X Child-Centered Practices	−.15	.14	.144
b			
Predictors for Children’s Restudy Effectiveness	*β* (standardized)	SE	*p* value
Intercept	.04	.09	.700
Grade (reference group 4th grade)	−.29	.12	.015
Teacher-Directed Practices	−.17	.08	.038
Child-Centered Practices	−.10	.07	.172
Grade X Teacher-Directed Practices	−.17	.10	.103
Grade X Child-Centered Practices	.32	.13	.021
c			
Predictors for Children’s Self-Monitoring Accuracy	*β* (standardized)	SE	*p* value
Intercept	.06	.09	.543
Grade (reference group 4th grade)	−.02	.13	.903
Teacher-Directed Practices	−.26	.08	.001
Child-Centered Practices	.23	.09	.019
Grade X Teacher-Directed Practices	−.23	.16	.150
Grade X Child-Centered Practices	−.13	.15	.399

*Note.* The standardized regression coefficients, standard errors of the standardized regression coefficients, and *p*-values indicating the significance levels of the regression coefficients for the effects of teachers’ instructional practices (teacher-directed and child-centered) on children’s (a) task performance, (b) restudy effectiveness, and (c) self-monitoring accuracy. *p < .05; **p < .01

For effectiveness of restudy, however, results depicted in Table 5b show a different effect of teacher-directed instructions. That is, the frequency of teacher-directed instructions was negatively related to children’s ability to strategically select the items for restudy for which their responses were incorrect. For child-centered instructions, there was no significant main effect on restudy. However, there was an interaction effect between grade and child-centered instructions, such that for second graders, child-centered instruction was more strongly and positively related to restudy effectiveness than for fourth graders.

Moreover, as shown by Table 5c, also for self-monitoring accuracy, there was a negative effect of the number of teacher-directed instructions, such that more teacher-directed instruction was related to less accurate discrimination between correct and incorrect task responses when children made CJs. Interestingly and in contrast to teacher-directed instructions, child-centered instructions were positively related to children’s self-monitoring accuracy. That is, in classrooms where teachers gave more child-centered instructions, children more accurately discriminated between well-learned and less-learned items.

## Discussion

In the elementary school years, children’s cognitive and metacognitive skills develop extensively. This age-related improvement seems to be caused not only by maturational, but also by contextual factors, and schooling may be one of the most important contextual factors. Much remains unclear about how classroom factors are linked to children’s metacognitive accuracy; the present research therefore aimed to investigate this. Particularly, contextual features of the classroom were assessed by investigating the content of teachers’ strategy instructions (i.e., whether they talked about specific cognitive and metacognitive strategies), and by investigating the framing of instructions about strategy use (i.e., whether instructions were delivered in a teacher-directed or in a child-centered way).

Previous research indicates effects of teacher instructions on elementary school children’s strategy use and cognitive development (e.g., Grammer et al. 2013; Ornstein et al. 2010). However, in these previous studies, no standardized measures were used to assess children’s achievement and procedural metacognition in direct relation to the instructed content. A unique contribution of the present research is that this design enables us to directly investigate relations between teacher instructions and children’s metacognitive accuracy and learning. Because all participating teachers instructed the same secret code task materials to children in their classroom in their own way, we could compare teachers. Further, children were tested with standardized materials assessing memory for the secret code materials, as well as procedural metacognition (measured with effectiveness of restudy selections and self-monitoring accuracy of confidence judgments). To investigate how teacher instructions may depend on children’s age, both second and fourth grade elementary school teachers and their school classes were included.

### Teachers’ strategy instructions and instructional practices

When instructing cognitive strategies, teachers told children about helpful techniques and tricks to accomplish the secret code task. When instructing metacognitive strategies, teachers discussed approaches children could use for planning, self-monitoring during learning, and self-evaluation after learning the secret code. Teachers seemed to more often instruct about cognitive than metacognitive strategies, which confirms previous classroom observation research (Dignath and Büttner 2018; Spruce and Bol 2015). Moreover, although teachers discussed cognitive and metacognitive strategies, modelled these, asked questions about these, and told children to practice these in the classroom, in most of these cases, teachers did not explicitly explain and evaluate why the particular strategies were useful. This confirms findings by Dignath-Van Ewijk et al. (2013) that teachers rarely elaborate on the instructed strategies and evaluate its usefulness.

Interestingly, it seems that teachers more often instructed about metacognitive strategies than teachers in classroom observation studies by Dignath and Büttner (2018) and Spruce and Bol (2015). They found that, although teachers expressed positive beliefs about teaching for metacognition, actual teaching of metacognitive strategies was low. Teachers rather seem to focus more on task content (McNamara 2010). A reason for this could be that teachers do not receive enough training on how to support their students with metacognition (Dignath and Büttner 2018). Surprisingly, in the present study teachers often instructed about metacognitive strategies related to planning, monitoring, and evaluation. The fact that the teachers all volunteered to be part of a larger research project investigating children’s metacognitive development, may have led them to focus their attention more on teaching metacognitive strategies than teachers in other classroom observation studies. Further, for the secret code task used in the present study, learning goals were very clear, which is hardly the case in everyday teaching. Thus, instructing metacognitive strategies might have been easier than in previous studies in which more naturalistic classroom situations were observed and coded.

Moreover, although both teacher-directed and child-centered teaching practices were observed in all classrooms, as expected, teachers most often instructed in a teacher-directed manner (cf. Alford et al. 2016; Stright and Supplee 2002). That is, most often they explicitly explained and modeled how to work on the task, rather than giving children autonomy and opportunities to self-regulate their learning of the task by themselves or in small groups. Furthermore, as hypothesized and as demonstrated by prior classroom observation research (Dignath and Büttner 2018; Ornstein et al. 2010), there was extensive variability between teachers in the extent to which they instructed about strategies, and used teacher-directed and child-centered practices. That is, some teachers almost never discussed strategies whereas other teachers often talked about this, and although all teachers employed teacher-directed and child-centered practices, there was substantial variance in the balance between these two types of teaching practices.

### Relations between classroom instructions and Children’s performance and metacognition

For children, findings firstly showed that fourth graders had better memory for the secret code task items than second graders. Furthermore, fourth graders made more effective restudy selections; in comparison to second graders, they more often selected items for which responses were incorrect. For self-monitoring accuracy, no age differences were found. This aligns with research showing that when learning paired-associates, developmental differences are most pronounced for effectiveness of regulation of study, whereas children’s monitoring development does not seem to undergo strong developmental changes after the age of eight (Schneider and Löffler 2016).

Importantly, findings showed that children’s performance, restudy selections, and self-monitoring were affected by the classroom they were in. This study provides us with the unique opportunity to relate variability in cognitive and metacognitive strategy instructions and variability in teacher-directed and child-centered instructional practices at the classroom level to children’s memory and procedural metacognition. Firstly, for children’s memory performance, findings show that in classrooms where teachers more often instructed about cognitive strategies, children had better memory for the secret code items. Considering the rapid changes in cognitive development in the elementary school years (Bjorklund and Causey 2017), this improved performance of fourth graders in comparison to second graders does not seem surprising. Moreover, children become more effective in application of strategies when they grow older, and further, children become better able to metacognitively evaluate whether strategies are useful (Imbo and Vandierendonck 2007; Schneider and Löffler 2016). These age-related improvements seem to be due to increases in cognitive capacity (e.g., working memory; Imbo and Vandierendonck 2007), as well as experiences made with educational tasks (Roebers et al. 2019). This may explain why fourth graders seemed to benefit more from teaching of cognitive strategies than second graders.

Moreover, also when assessing self-monitoring accuracy, there was a positive relation between teaching cognitive strategies and the accuracy of children’s CJs. These findings contribute to the debate on causes of development of memory, and seem to imply that children’s development of memory and metacognition is partially due to their more effective use of instructed strategies. Further, these findings seem to indicate that teachers’ instructional style plays an important role for children’s cognitive and metacognitive development. Findings on relations between cognitive strategy teaching and performance (measured with an immediate memory test) are not surprising; research on complex math learning (Zepeda et al. 2019) as well as long-term studies with use of transfer tasks showed that teachers’ strategy instructions are linked to cognitive performance (Grammer et al. 2013; Ornstein et al. 2010). For metacognition, until present, research only documented relations between teachers’ strategy instructions and children’s cognitive and metacognitive strategy use (Grammer et al. 2013; Leidinger and Perels 2012; Ornstein et al. 2010). The present study is the first to show that cognitive strategy instruction is positively related to children’s self-monitoring accuracy.

However, for relations between cognitive strategy instructions and children’s metacognitive control, as measured with restudy selections, findings show a contrasting picture; the relation between cognitive strategy teaching and effective restudy was negative, rather than positive. At first sight, this finding seems surprising. However, this may indicate that not only the content of the instructions itself, but specifically instructional practices, are related to children’s metacognition. Explicit investigation of the overlap between cognitive and metacognitive strategy instructions and child-centered and teacher-directed instructional practices goes beyond the scope of this research. However, Appendix Table 8 has been included to give the reader insight into potential overlap between these codes; there seems to be extensive overlap between coding for cognitive strategies and coding for teacher-directed instructional practice. This may imply that when teaching about cognitive strategies, teachers only provided children with limited opportunities and autonomy to self-regulate their learning, and to practice use of these strategies. Even though teaching about cognitive strategies gave children better insights into well-learned and less-learned items (as measured with their self-monitoring judgments), the teacher-directed practice of strategy teaching may not have improved children’s motivation to further engage with the learning task, and to restudy task items that were not yet well learned (Stright and Supplee 2002).

Also metacognitive strategies seem to be more often instructed in a teacher-directed, rather than in a child-centered way. Particularly for young elementary school children, it may be challenging to apply metacognitive strategies. Teachers may have considered it necessary to give students clear guidance about how to use metacognitive strategies. However, the frequency of instruction for metacognitive strategies was not related to children’s performance and metacognition. Possibly, students would be more willing to take up strategy instructions when these would be delivered through child-centered, rather than teacher-directed practices.

Our analyses on relations between teacher-directed and child-centered practices and children’s memory and metacognition provide further evidence for this assumption. Both styles of teacher practices seem to support children’s task performance. Presumably, the teacher-directed instructions helped children to work on the task in an organized manner (Stright and Supplee 2002), whereas the child-centered instructional practices may have benefitted children’s motivation to work on the task, and their attention focusing and behavioral engagement (Lerkkanen et al. 2016; Rimm-Kaufman et al. 2009; Stright and Supplee 2002).

However, although teacher-directed instruction may support children to memorize task items, our findings show that a higher frequency of teacher-directed instructions was related to less effective restudy and less accurate self-monitoring. Child-centered, rather than teacher-directed instructions seemed most beneficial to foster children’s metacognition in the classroom. Importantly, for second graders, child-centered teaching was positively related to the effectiveness of restudy selections. One reason why child-centered instructions were positively related to the effectiveness of restudy for second, but not for fourth graders, could be due to the strong developmental improvement in children’s metacognitive control during the early elementary school years (Roebers 2017). The present research confirms that fourth graders made more effective restudy selections. Possibly, for second graders, there was room to further develop effective control skills, and these may have accelerated when teachers provided them with opportunities to practice self-regulation of learning in the classroom. The finding that we did not find relations between child-centered teaching and effective restudy for fourth graders may indicate that they reached their maximum potential for effective control with the present task (even though fourth graders selected only less than half of the incorrect items for further study). Importantly, child-centered instructions were positively related to self-monitoring accuracy for both grades. This seems to indicate that giving children opportunities to practice self-regulation when working on the task helped them to monitor what had been well learned and what not.

### Limitation and directions for future research

Even though most teachers regularly discussed metacognitive strategies, there was no relation between teaching metacognitive strategies and children’s performance and metacognition. These findings seem surprising, since meta-analyses by Donker et al. (2014) and Dignath et al. (2008) showed that emphasizing metacognitive strategies positively affected long-term achievement for primary school children. However, it has to be noted that the secret-code learning task that teachers were instructing in this study may not be representative of typical classroom learning tasks. Teachers particularly focused on association strategies to connect the secret code elements to the letters of the alphabet. When teaching about actual educational tasks, instruction can only be effective when it builds on conceptual and prior knowledge (e.g., Ozuru et al. 2009). However, for the secret code task, which was selected such that it was novel for teachers as well as children, building on prior knowledge was not a necessity. This may be a reason why for this novel memory-based task, instructions about metacognitive strategies were not related to children’s test performance and metacognition. Moreover, although for this task, particularly associating elements seemed to be an effective cognitive strategy, for more complex and educationally embedded learning tasks, not only one specific strategy, but rather a combination of multiple strategies would seem helpful. That is, metacognitive strategies like planning and monitoring would be necessary to obtain good performance, and instruction of metacognitive strategies would likely affect children’s cognitive and metacognitive outcomes. Future research should further investigate how connections between metacognitive strategy instructions and children’s metacognition and learning depend on task characteristics.

Paired-associate learning tasks are frequently used in research on children’s procedural metacognition (Schneider and Löffler 2016), and by using these associative learning materials, we were able to base conclusions on well-validated behavioral measures of self-monitoring accuracy and restudy effectiveness. For a first study on relations between teachers’ instructions and children’s metacognitive accuracy, we considered it a necessity to use these reliable and replicable measures. However, these measures also come with limitations. Measurement took place immediately after teachers instructed the task, whereas in the educational context, it is most interesting to investigate whether teacher instructions improve long-term learning and transfer. Although prior research showed positive long-term effects of teacher instructions on children’s memory (Ornstein et al. 2010), future research should address long-term relations between classroom instructions and children’s metacognition.

Further, we expected that teachers would more often use child-centered teaching for fourth than second graders, based on the assumption that older children are better able to deal with autonomy, and that teachers would adapt their teaching practices accordingly (Collins 1996; Perry et al. 2015). However, findings for second and fourth grade teachers were highly similar, both for the frequency of teacher-directed and child-centered instructions, and for the frequency of cognitive and metacognitive strategy instructions. On the one hand, these observations of similarities between grades may strengthen our findings, since these may provide us with a within-study replication. On the other hand, however, it has to be noted that our sample size for teachers was small, only 21 teachers participated. Further research is needed to draw more definite conclusions about how teachers may adapt their instructions to the grade levels they are teaching, and how this may depend on the type of learning task. Moreover, beyond children’s age, further child characteristics (for instance intelligence and working memory capacity) are likely to affect the relation between teacher instructions and children’s cognitive and metacognitive outcomes. Future research should attempt to include such measures of individual differences between children as covariates.

Finally, teacher utterances could receive multiple codes, and there was overlap in coding for cognitive and metacognitive strategy instructions and teacher-directed and child-centered teaching (as shown in Appendix Table 8). This may indicate that there was a connection between strategy teaching and teaching practices. Although very novel and interesting, these findings need to be interpreted with caution. This overlap may be affected by the nature of our task, and may not necessarily be directly translatable to teaching of more complex problem solving and comprehension tasks. Future research should therefore investigate whether these findings on the relation between cognitive and metacognitive talk in teacher-directed versus child-centered practices are replicable.

## Conclusions

The present research shows connections between teachers’ instructions and children’s immediate memory and metacognition. Particularly, the observed individual differences between children in performance, restudy effectiveness, and self-monitoring accuracy could in part be attributed to classroom instructions. Findings seem to indicate that teachers’ cognitive strategy instructions, as well as teacher-directed and child-centered instructional practices, are positively related to children’s memory performance. However, for metacognition, child-centered classroom practices, rather than the content of instructional support per se, seems to be associated with more accurate self-monitoring accuracy. These findings may indicate that strategy instruction is mainly beneficial when children are at the center of instruction. Moreover, for second grade children, when selecting items for restudy, findings may imply that through child-centered teaching, children’s attention can be focused on the opportunities to self-regulate their learning. In sum, these research findings capture some of the complexity of the processes underlying children’s (meta)cognitive development, and indicate that teachers’ contextual influences should not be neglected.

## Figures and Tables

**Table 7:**
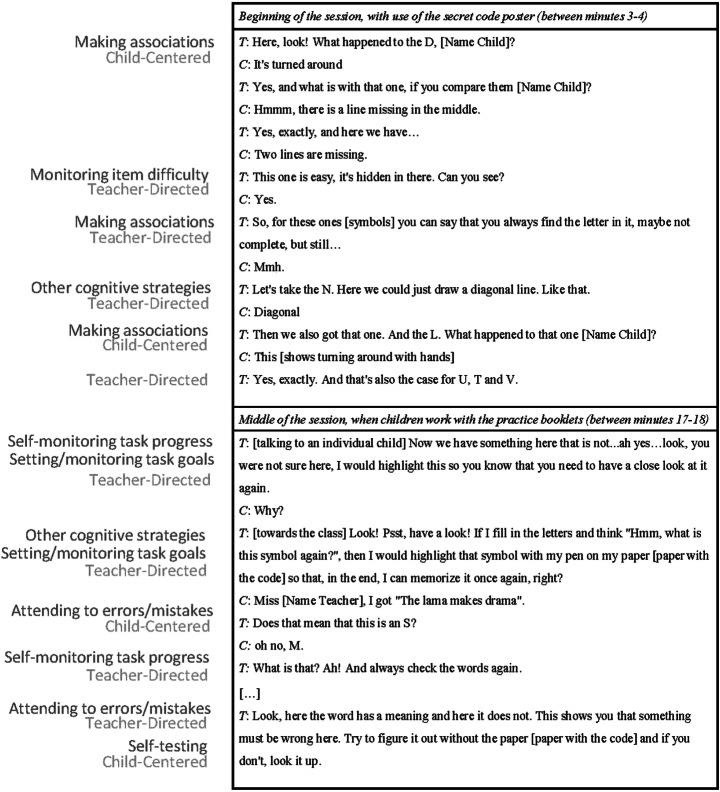
Example of coding of a transcript

Part of a transcript (translated from [XXX]) and coding of teacher strategy instructions and instructional practices

## Appendix

**Table 6:** Frequency count of individual codes for strategy instructions

	Grade 2 Teachers		Grade 4 Teachers		Overall
	Mean	Range	Mean	Range	Mean
Cognitive Strategies					
Categorizing and Making Associations	30.78	18–54	35.08	8–71	33.24
Testing	13.00	0–37	12.00	2–32	12.43
Other cognitive strategies	3.33	1–5	2.67	0–5	2.95
Metacognitive Strategies					
Goal setting and reminding about goals	3.89	0–13	4.33	0–9	4.14
Planning time and use of materials	3.89	1–7	4.75	1–13	4.38
Paying attention to item difficulty	9.67	0–19	8.58	0–17	9.05
Self-monitoring progress and task understanding	10.44	2–28	7.17	2–18	8.57
Explaining and evaluating strategy use	5.22	0–19	7.50	0–19	6.53
Discussing errors and mistakes	3.22	0–12	2.50	0–12	2.80

The frequency count and the range for the individual codes for strategy instructions for both grades. For analyses, these codes were collapsed to reflect the overall frequency count of teaching for cognitive and metacognitive strategies

**Table 8:** Overlap between codes

	Overlap in coding (frequencies and percentages)			
Instructional Language	Cognitive Strategies	Metacognitive strategies	Teacher-Directed Practices	Child-Centered Practices
Cognitive Strategies		104 (10.12%)	514 (50.00%)	410 (39.88%)
Metacognitive Strategies	104 (12.86%)		486 (60.07%)	219 (27.07%)
Teacher-Directed Practices	514 (50.44%)	486 (47.69%)		19 (1.86%)
Child-Centered Practices	410 (63.27%)	219 (33.80%)	19 (2.93%)	

Multiple codes could be assigned to the same unit of teachers’ instructional language. The table shows, in case of overlap between codes, how often codes co-occurred (including the row-wise percentages)
